# MFS transporters required for multidrug/multixenobiotic (MD/MX) resistance in the model yeast: understanding their physiological function through post-genomic approaches

**DOI:** 10.3389/fphys.2014.00180

**Published:** 2014-05-08

**Authors:** Sandra C. dos Santos, Miguel C. Teixeira, Paulo J. Dias, Isabel Sá-Correia

**Affiliations:** Institute for Biotechnology and Bioengineering, Centre for Biological and Chemical Engineering, Department of Bioengineering, Instituto Superior Técnico, Universidade de LisboaLisbon, Portugal

**Keywords:** *Saccharomyces cerevisiae*, multidrug/multixenobiotic resistance (MDR/MXR), MDR/MXR transporters, Major Facilitator Superfamily (MFS), genome-wide approaches, transcriptional regulation, phylogenetic analysis

## Abstract

Multidrug/Multixenobiotic resistance (MDR/MXR) is a widespread phenomenon with clinical, agricultural and biotechnological implications, where MDR/MXR transporters that are presumably able to catalyze the efflux of multiple cytotoxic compounds play a key role in the acquisition of resistance. However, although these proteins have been traditionally considered drug exporters, the physiological function of MDR/MXR transporters and the exact mechanism of their involvement in resistance to cytotoxic compounds are still open to debate. In fact, the wide range of structurally and functionally unrelated substrates that these transporters are presumably able to export has puzzled researchers for years. The discussion has now shifted toward the possibility of at least some MDR/MXR transporters exerting their effect as the result of a natural physiological role in the cell, rather than through the direct export of cytotoxic compounds, while the hypothesis that MDR/MXR transporters may have evolved in nature for other purposes than conferring chemoprotection has been gaining momentum in recent years. This review focuses on the drug transporters of the Major Facilitator Superfamily (MFS; drug:H^+^ antiporters) in the model yeast *Saccharomyces cerevisiae*. New insights into the natural roles of these transporters are described and discussed, focusing on the knowledge obtained or suggested by post-genomic research. The new information reviewed here provides clues into the unexpectedly complex roles of these transporters, including a proposed indirect regulation of the stress response machinery and control of membrane potential and/or internal pH, with a special emphasis on a genome-wide view of the regulation and evolution of MDR/MXR-MFS transporters.

## Multidrug/multixenobiotic transporters of the major facilitator superfamily in the model yeast *Saccharomyces cerevisiae*

### The multidrug/multixenobiotic resistance phenomenon

All living cells exhibit elaborate molecular mechanisms to protect themselves from external assault, such as the exposure to drugs and other cytotoxic compounds. The simultaneous acquisition of resistance to a wide range of structurally and functionally unrelated cytotoxic chemicals has become a widespread phenomenon in nature known as Multidrug/Multixenobiotic resistance (MDR/MXR) (Gulshan and Moye-Rowley, 2007; Higgins, 2007; Nikaido, 2009; Sá-Correia et al., 2009). MDR/MXR poses a severe problem at the clinical and agricultural levels, being involved in the failure of cancer therapy and treatment of infectious diseases, as well as food preservation and crop protection strategies, due to the emergence of resistant cancer cell lines, pathogenic strains, and resistant weed species (Prasad and Kapoor, 2005; De Waard et al., 2006; Szakács et al., 2006; Hall et al., 2009; Morschhauser, 2010).

Although drug resistance might be mediated by various mechanisms, membrane transporters that presumably catalyze the efflux of a broad range of cytotoxic substrates play an important role in MDR/MXR processes (Sá-Correia et al., 2009; Sherlach and Roepe, 2014). Such MDR/MXR pumps can presumably recognize a wide variety of unrelated compounds that are usually not present in the cell's natural environment, maintaining the intracellular drug/xenobiotic concentration below its toxic level and thus providing chemoprotection from those compounds (del Sorbo et al., 2000; Higgins, 2007; Prasad and Goffeau, 2012). Several MDR/MXR families of transporters have been identified and studied in different types of cells, however only two are known to occur commonly in all classes of organisms: the ATP-binding cassette (ABC) superfamily and the Major Facilitator Superfamily (MFS) (Higgins, 2007; Sá-Correia et al., 2009). MFS transporters, the focus of this review, are ubiquitous single-polypeptide secondary carriers capable only of transporting small solutes in response to chemiosmotic ion gradients. However, in some cases, MFS transporters are also believed to act as Drug:H^+^ Antiporters (DHA) and confer MDR/MXR resistance in bacteria, fungi and more complex eukaryotes.

MDR/MXR transporters have puzzled researchers for a long time, but their refractoriness to purification and crystallization has handicapped the elucidation of the mechanisms behind their action. Plus, the genome of one single cell can encode multiple MDR/MXR transporters, some of which are apparently redundant (Paulsen, 2003; Higgins, 2007). The high number of these transporters is a clear indication that MDR/MXR mediated by efflux pumps is not an exceptional phenomenon, but a highly conserved defense mechanism. Importantly, most multidrug transporters share significant homology with other transporters with well-characterized natural substrates (Paulsen, 2003). As such, many questions still await answers, one of the most important pertaining to the normal physiological function of these so-called “drug efflux pumps” (Roepe, 2000; Panwar et al., 2008; Sá-Correia et al., 2009; Sherlach and Roepe, 2014): do they only offer protection from cytotoxic agents, or did they evolve in nature for other purposes? Do they have relevant physiological substrates and only extrude toxic compounds opportunistically? Is their role in drug resistance effectively connected to direct drug transport, as has been believed for years, or do they instead have an indirect action in MDR/MXR?

Although the molecular mechanisms underlying the apparent promiscuity of MDR/MXR transporters have remained a long-standing topic of debate, recent findings, also derived from genome-wide strategies, are shifting the discussion toward an indirect role of, at least, some of these transporters in MDR/MXR, in contrast to the more widely accepted drug-efflux pump model (Vargas et al., 2007; Cabrito et al., 2011; Teixeira et al., 2011; Dikicioglu et al., 2013; Kruger et al., 2013). Moreover, several recent analyses have highlighted the correlation between MDR/MXR and membrane lipid homeostasis (Pallares-Trujillo et al., 2000; Panwar et al., 2008; Shahi and Moye-Rowley, 2009). The coordinate control of lipid composition and drug transport activities is required for normal multidrug resistance in fungi and, indeed, the regulation of membrane composition by MDR/MXR proteins provides an important contribution to the resistant phenotypes observed. The altered partitioning model is another interesting hypothesis that attempts to explain the mode of action of MDR/MXR transporters (Roepe et al., 1996; Sherlach and Roepe, 2014). It suggests that MDR/MXR proteins do not directly transport drugs, but instead their altered expression results in the alteration of key parameters, which ultimately leads to changes in the passive diffusion and/or action of cytotoxic compounds. Understanding the exact physiological role of these transporters, and how it relates to their action in MDR/MXR, is of paramount importance to devise strategies to overcome clinical multidrug resistance, achieve a more efficient uptake and utilization of drugs, and improve stress tolerance in biotechnological processes.

This review will focus on the physiological characterization of MFS transporters involved in MDR/MXR in the model yeast, and how the proposed physiological roles might be associated with chemical stress resistance. We also discuss the potential of genome-wide approaches, not only in the elucidation of specific biological roles but also in the unraveling of mechanisms of transcriptional and post-translational control of the activity of these transporters.

### *S. cerevisiae* as an experimental model in MDR/MXR

Yeast is a widely used eukaryotic model for molecular and cellular biology studies. This unicellular non-pathogenic microorganism is a robust and inexpensive experimental platform, amenable to genetic manipulation, possesses a remarkable level of functional conservation with higher eukaryotes, and its genome has been extensively annotated with functional information. But even more significantly, yeast has been used to pioneer the development of several post-genomic experimental approaches and computational tools, allowing the easy implementation of genome-wide analyses and the availability of a wide range of experimental tools and biological material (Mager and Winderickx, 2005; Smith et al., 2010; Botstein and Fink, 2011). Moreover, although many cytotoxic compounds of interest do not exist in the natural environment of yeast, many of the basic mechanisms underlying adaptation and resistance to chemical and other environmental stresses are apparently conserved between yeast and phylogenetically distant organisms. As such, the use of this model system can provide a deep level of understanding on molecular mechanisms that would be harder to achieve in more complex and less accessible eukaryotes. Furthermore, the results emerging from studies on MDR/MXR and its regulation in *S. cerevisiae* can be extended to pathogenic yeasts (e.g., *Candida* species) to guide the development of new prophylactic, diagnosis, and therapeutic approaches to the rising number of drug resistant fungal infections (see Costa et al. in this Research Topic and refs Costa et al., 2013a,b, 2014).

The use of genome-wide (*Omic*) approaches has gained considerable momentum in recent years. Remarkably, the identification of alterations occurring in gene or protein expression following exposure to a drug or xenobiotic compound can contribute to identify the cellular pathways that are most relevant to the stress response, including MDR/MXR transporters and how their complex regulation and putative physiological roles may also influence drug resistance. Genomic/proteomic variations can be identified through transcriptomics and quantitative proteomics. Expression proteomic approaches are also important to identify alterations at the level of post-translational modifications associated with exposure to xenobiotic and cytotoxic compounds. Another very important tool in yeast research are gene knockout collections. Chemogenomic analyses to identify determinants of resistance to a particular compound in yeast can be performed using homozygous (knock-out deletions, gene dosage = 0%), haploinsufficiency (heterozygous deletion strains, gene dosage = 50%) and multicopy- and overexpression (gene dosage > 100%) collections. Many MDR/MXR transporters are directly linked to the ability of cells to grow in the presence of a particular stressing condition, and most of the compounds to which these transporters provide protection have been identified through the assessment of the fitness phenotypes of the corresponding deletion mutants under chemical stress. In fact, under control conditions these growth phenotypes are not detectable and would not have been identified. Furthermore, in several cases the presence of the gene is required for growth under a given stress, and yet there are no alterations at the expression or protein content level, meaning that many of these conditions would not have been discovered using expression-based methodologies such as DNA microarrays or proteomics. However, the identification of an MDR/MXR transporter-encoding gene as a determinant of resistance does not necessarily mean that the gene product is involved directly in the efflux of that compound from the cell. As discussed in Section The MDR/MXR Phenomenon, some of these transporters might have natural physiological roles that indirectly enable chemoprotection, for example through the alteration of plasma membrane potential and/or lipid composition. For this reason, lipidomic analyses can also be of particular interest, given the role of sphingolipids and ceramides as signaling mediators in a growing number of pathways and in lipid-based signaling in the yeast response to stress (Epstein and Riezman, 2013), in addition to the effect of plasma membrane ergosterol content and membrane lipid homeostasis in stress tolerance and drug resistance (Panwar et al., 2008; Shahi and Moye-Rowley, 2009; Cabrito et al., 2011).

The identification of transcription factors and regulatory networks predicted to underlie the transcriptional regulation of MDR/MXT transporters has been facilitated by freely accessible databases, such as YEASTRACT (http://www.yeastract.com) (Teixeira et al., 2006, 2014). This information system contains over 200,000 regulatory associations between transcription factors and target genes, including 326 DNA binding sites for 113 transcription factors. The most recent version includes further information on the environmental conditions in which each association was determined and whether the transcription factor is acting on its target genes as activator or repressor, enabling the development of a wide range of new queries (Teixeira et al., 2014). More in-depth studies into the physiological activity of MDR/MXR-MFS transporters can be designed based on the knowledge obtained from the transcriptional networks governing the expression of these transporters in response to specific stress conditions. Some examples retrieved from the YEASTRACT database are discussed in Section Clues on the Functional Analysis of DHA1/DAG Transporters Based on their Transcriptional Regulation Under Environmental Challenges or Stress.

### The MDR/MXR-related subfamilies of MFS transporters DHA1 and DAG in *S. cerevisiae*

**Figure 1:**
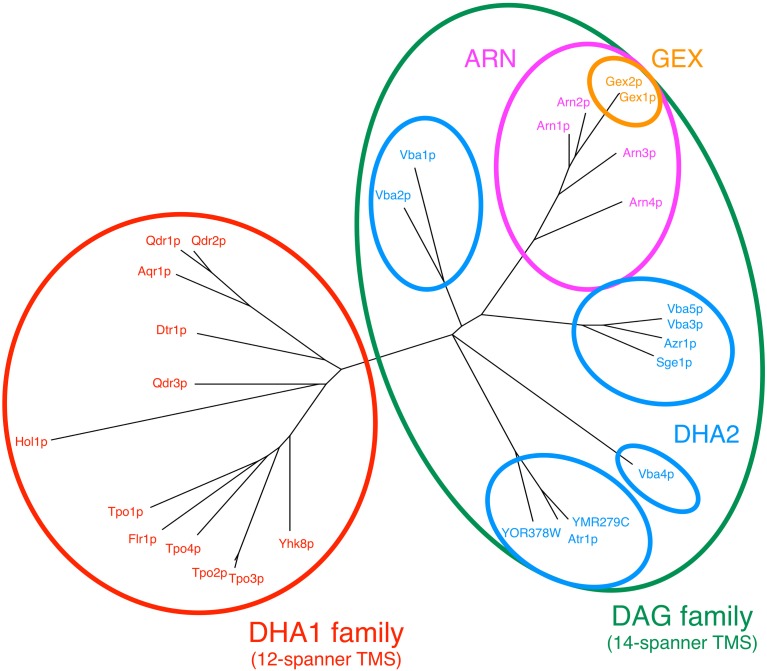
**Phylogenetic tree constructed using the amino acid sequences of DHA1 and DAG proteins encoded in the genome of the model-organism *S. cerevisiae***. The multiple alignments of the protein sequences were done using Muscle software and the tree was built using the maximum likelihood package (PROTML) made available in the PHYLIP software suite (Felsenstein, 1989; Edgar, 2004).

Following the release of the full genome sequence of *S. cerevisiae* (Goffeau et al., 1996), the similarity analysis of the amino acid sequences encoded in the annotated ORFs led to the separation of MDR/MXR-MFS encoded genes into 2 sub-families, depending on whether their protein products contained 12 or 14 transmembrane segments (TMS): the 12-spanner drug:H^+^ antiporter family 1 (DHA1) and the 14-spanner drug:H^+^ antiporter family 2 (DHA2) (Nelissen et al., 1995, 1997). The subsequent phylogenetic analysis of these protein sequences showed that they fell into three major clusters, with cluster I comprising the 12-spanner MDR/MXR-MFS transporters and clusters II and III comprising the 14-spanner transporters. While cluster II included the DHA2 family proteins, those in cluster III were assigned to the Unknown Major Facilitator (UMF) family (Paulsen et al., 1998). However, after the demonstration that four UMF family members encoded siderophore transporters (Lesuisse et al., 1998; Heymann et al., 1999, 2000a,b) and that the other two UMF family members encoded glutathione exchangers (GEX) (Dhaoui et al., 2011), these proteins were reassigned to the new ARN (also known as the Siderophore-Iron Transporter/SIT family) and GEX families, respectively (Yun et al., 2000; Haas et al., 2008; Dhaoui et al., 2011; Dias and Sa-Correia, 2013). However, a recent analysis combining phylogenetic and gene neighborhood approaches gathered evidence supporting the hypothesis that the DHA2, ARN and GEX proteins share a common root (Dias and Sa-Correia, 2013). A new gene family, DAG (DHA2/ARN/GEX), was proposed to accommodate these three phylogenetic subfamilies of 14-spanner MFS transporters (Figure 1; Tables 1, 2).

**Table 1:** **List of MDR/MXR-MFS transporters encoded in the genome of *S. cerevisiae*, belonging to the DHA1 family, gene product descriptions are based on the information listed for each gene in the *Saccharomyces* Genome Database**.^**a**^

**Gene**	**Name**	**Gene product description**
*YNL065w*	*AQR1*	Confers resistance to short-chain monocarboxylic acids and quinidine; involved in the excretion of excess amino acids; has a paralog, *QDR1*, that arose from the whole genome duplication; relocalizes from plasma membrane to cytoplasm upon DNA replication stress; boron exporter
*YBR180w*	*DTR1*	Putative dityrosine transporter; required for spore wall synthesis; sequence similarity to *QDR1* and *QDR3*, and the triple mutant *dtr1 qdr1 qdr3* exhibits reduced dityrosine fluorescence relative to the single mutants; expressed during sporulation
*YBR008c*	*FLR1*	Involved in the efflux of fluconazole, diazaborine, benomyl, methotrexate, and other drugs; expression is induced in cells treated with the mycotoxin patulin; relocalizes from nucleus to plasma membrane upon DNA replication stress
*YNR055C*	*HOL1*	Mutations in membrane-spanning domains permit cation and histidinol uptake
*YIL120w*	*QDR1*	Involved in spore wall assembly; sequence similarity to *DTR1* and *QDR3*, and the triple mutant *dtr1 qdr1 qdr3* exhibits reduced dityrosine fluorescence relative to the single mutants; required for resistance to quinidine, ketoconazole, fluconazole, and barban; has a paralog, *AQR1*, that arose from the whole genome duplication
*YIL121w*	*QDR2*	Exports and is regulated by copper; has broad substrate specificity and can transport many mono- and divalent cations; transports a variety of drugs and is required for resistance to quinidine, barban, cisplatin, and bleomycin; contributes to potassium homeostasis
*YBR043c*	*QDR3/AQR2*	Has a role in polyamine homeostasis; involved in spore wall assembly; sequence similarity to *DTR1* and *QDR1*, and the triple mutant *dtr1 qdr1 qdr3* exhibits reduced dityrosine fluorescence relative to the single mutants; expression is up-regulated under polyamine stress; required for resistance to quinidine, barban, cisplatin, and bleomycin
*YLL028w*	*TPO1*	Polyamine transporter that recognizes spermine, putrescine, and spermidine; catalyzes uptake of polyamines at alkaline pH and excretion at acidic pH; phosphorylation enhances activity and sorting to the plasma membrane
*YGR138c*	*TPO2*	Polyamine transporter, specific for spermine; localizes to the plasma membrane; transcription is regulated by Haa1p; has a paralog, *TPO3*, that arose from the whole genome duplication
*YPR156c*	*TPO3*	Polyamine transporter, specific for spermine; localizes to the plasma membrane; has a paralog, *TPO2*, that arose from the whole genome duplication
*YOR273c*	*TPO4*	Polyamine transporter that recognizes spermine, putrescine, and spermidine; localizes to the plasma membrane
*YHR048w*	*YHK8*	Expression is up-regulated in cells exhibiting reduced susceptibility to azoles

a *For a comprehensive list of compounds to which each gene confers resistance (see Sá-Correia et al., 2009) and the more recent literature compiled for each gene in the Saccharomyces Genome Database*.

**Table 2:** **List of MDR/MXR-MFS transporters encoded in the genome of *S. cerevisiae*, belonging to the DAG family, gene product descriptions are based on the information listed for each gene in the *Saccharomyces* Genome Database**.^**a**^

**Gene**	**Name**	**Gene product description**
**DHA2 SUBFAMILY**		
*YML116w*	*ATR1/SNQ1*	Required for resistance to aminotriazole and 4-nitroquinoline-N-oxide; has a paralog, *YMR279C*, that arose from the whole genome duplication; putative boron exporter; protein abundance increases in response to DNA replication stress
*YGR224w*	*AZR1*	Involved in resistance to weak acids and azole drugs such as ketoconazole and fluconazole
*YPR198w*	*SGE1/NOR1*	Acts as an extrusion permease; partial multicopy suppressor of *gal11* mutations
*YMR088c*	*VBA1*	Permease of basic amino acids in the vacuolar membrane
*YBR293w*	*VBA2*	Permease of basic amino acids in the vacuolar membrane
*YCL069w*	*VBA3*	Permease of basic amino acids in the vacuolar membrane; has a paralog, *VBA5*, that arose from a segmental duplication
*YDR119w*	*VBA4*	Proposed role as a basic amino acid permease based on phylogeny; GFP-fusion protein localizes to vacuolar membrane; physical interaction with Atg27p suggests a possible role in autophagy
*YKR105c*	*VBA5*	Involved in amino acid uptake and drug sensitivity; has a paralog, *VBA3*, that arose from a segmental duplication
*YOR378w*		Putative paralog of *ATR1* but not required for boron tolerance
*YMR279c*		Putative boron transporter involved in boron efflux and resistance; overexpression mutant but not null mutant displays boron tolerance phenotype; heat-induced gene; has a paralog, *ATR1*, that arose from the whole genome duplication
**ARN SUBFAMILY**		
*YHL040c*	*ARN1*	Transporter for siderophore-iron chelates; responsible for uptake of iron bound to ferrirubin, ferrirhodin, and related siderophores; protein increases in abundance and relocalizes to the vacuole upon DNA replication stress
*YHL047c*	*ARN2/TAF1*	Responsible for uptake of iron bound to the siderophore triacetylfusarinine C
*YRL065w*	*SIT1/ARN3*	Ferrioxamine B transporter; transcription is induced during iron deprivation and diauxic shift; potentially phosphorylated by Cdc28p
*YOL158c*	*ENB1/ARN4*	Endosomal ferric enterobactin transporter; expressed under conditions of iron deprivation; expression is regulated by Rcs1p and affected by chloroquine treatment
**GEX SUBFAMILY**		
*YCL073c*	*GEX1*	H^+^:glutathione antiporter; localized to the vacuolar and plasma membranes; imports glutathione from the vacuole and exports it through the plasma membrane; has a role in resistance to oxidative stress and modulation of the PKA pathway; has a paralog, *GEX2*, that arose from a segmental duplication
*YKR106w*	*GEX2*	H^+^:glutathione antiporter; localized to the vacuolar and plasma membranes; expressed at a very low level; potential role in resistance to oxidative stress and modulation of the PKA pathway; has a paralog, *GEX1*, that arose from a segmental duplication

a *For a comprehensive list of compounds to which each gene confers resistance (see Sá-Correia et al., 2009) and the more recent literature compiled for each gene in the Saccharomyces Genome Database*.

As in many other species, the prevalence and apparent redundancy of these transporters in *S. cerevisiae* raises many questions, most importantly what is the physiological function of these proteins in the absence of drugs or stresses to which they confer resistance? Although MDR/MXR-MFS genes are not essential, they are encoded in the genome, and maintain basal expression levels when cells are not exposed to any stress. However, the identification of the so-called physiological function of these transporters is not trivial. Multispanner membrane transporters are very difficult to purify, and reconstitution in lipid bilayer preparations for direct functional assays presents many difficulties (Ambudkar et al., 1998; Howard and Roepe, 2003). An additional drawback is that the elimination of a membrane transporter will often have an effect on the transport kinetics of other transporters in the same cell, further complicating the functional analysis of the corresponding deletion mutants. The phylogenetic analysis of MDR/MXR-MFS transporters might shed light on the physiological functions of these transporters, based on the idea that amino acid conservation might imply similar biological functions, and in turn help elucidate the molecular mechanisms that lead to multidrug resistance.

#### The DHA1 family

The DHA1 family in *S. cerevisiae* comprises 12 proteins, encoded by *AQR1*, *DTR1*, *FLR1*, *HOL1*, *QDR1*, *QDR2*, *QDR3*, *TPO1*, *TPO2*, *TPO3*, *TPO4*, and *YHK8* (Table 1) (Sá-Correia et al., 2009; Dias et al., 2010). The expression of MDR transporters of the MFS has been shown to confer resistance to a wide variety of chemical stresses, ranging from anticancer and antimalarial drugs to weak acids and herbicides (Sá-Correia et al., 2009). However, although these transporters have been mostly studied within the context of their role in multidrug resistance, physiological roles for many DHA1 proteins have been proposed in recent years. In some cases, as described below, these natural functions have been correlated with the transporter's ability to confer chemoprotection against a given stress, consistent with the hypothesis of an indirect role in drug resistance.

Dtr1 was one of the first DHA1 proteins that were shown to have a physiological role other than chemoprotection. This transporter was identified in a genome-wide screen for spore wall mutants and was shown to be essential for its biosynthesis, by facilitating the translocation of bisformyl dityrosine through the prospore membrane during spore wall maturation (Felder et al., 2002). Bisformyl dityrosine was therefore proposed as a natural substrate of Dtr1, although the transporter was also shown to be a determinant of resistance to several cytotoxic compounds (Felder et al., 2002; Sá-Correia et al., 2009). Aqr1 was another one of the first DHA1 family transporters to have a physiological role assigned. This transporter has been demonstrated to be involved in the excretion of amino acids, in particular homoserine and threonine (Velasco et al., 2004), a role that might be particularly relevant in the response to stress conditions leading to an abnormal accumulation of amino acids. More recently, a genome-wide study of DNA damage pathways in response to DNA replication stress also found that Aqr1 relocalizes from the plasma membrane to the cytoplasm when cells are challenged with the replication inhibitor hydroxyurea, suggesting that Aqr1 might be involved in a pathway that responds to drug-induced DNA replication stress (Tkach et al., 2012). The same study also showed that Flr1 relocalizes from the nucleus (confirmed localization under control conditions, although it does not match the localization predicted by Huh et al., 2003) to the plasma membrane following treatment with methylmethanesulfonate (MMS), an alkylating agent that interferes with the action of replicative polymerases. This observation is of particular interest since Flr1 is one of the MDR/MXR-MFS transporters that is more clearly linked to drug resistance, enabling yeast cells with increased resistance to a wide range of unrelated drugs and other chemicals (Sá-Correia et al., 2009), but a physiological substrate has not yet been described. Although Qdr1 is a paralog of Aqr1, it was not implicated in the response to replication stress (Tkach et al., 2012), nor does it have a natural substrate assigned. However, a synthetic genetic array analysis recently identified Qdr1 as a component of the complex gene network that controls the assembly of the spore wall, together with Dtr1 and Qdr3 (Lin et al., 2013).

Qdr2 has been proposed to contribute to potassium homeostasis, possibly by functioning as an alternative K^+^ importer (Vargas et al., 2007). This physiological function was proposed to play an indirect role in the ability of this transporter to confer resistance to quinidine (Vargas et al., 2004, 2007), a drug that leads to decreased K^+^ uptake and a drop in the intracellular accumulation of K^+^ in yeast cells (Vargas et al., 2007). *QDR2* expression was shown to confer a physiological advantage to cells during the onset of K^+^ limited growth and in the presence of quinidine by promoting K^+^ import (Vargas et al., 2007). Qdr2 has also been isolated in a global screen for tolerance to lithium and sodium and was proposed to participate in the uptake of cadmium and cobalt (Rios et al., 2013). Furthermore, copper extrusion is impaired in the *qdr2* null mutant, leading to the proposal of divalent copper as the main physiological substrate of Qdr2. This role in copper homeostasis (an important substrate for several intracellular redox reactions) might also provide an explanation for the involvement of Qdr2 in the oxidative stress response (Rios et al., 2013). Interestingly, the Qdr2 homolog in *C. glabrata*, CgQdr2, although conferring resistance to the same drugs as its *S. cerevisiae* counterpart, was not found to confer an advantage to *C. glabrata* or *S. cerevisiae* cells grown under K^+^ limitation, nor did it complement the *S. cerevisiae qdr2* null mutant phenotype under these conditions (Costa et al., 2013a).

Qdr3, which is involved in spore wall assembly together with its close homologs Dtr1 and Qdr1 (Lin et al., 2013), was recently suggested to have a still undetermined role in respiration, with *qdr3* null mutants displaying a respiratory deficiency under glucose depletion (Dikicioglu et al., 2008). The same authors later employed an integrated transcriptomics and metabolomics approach to find that cells lacking Qdr3 (as well as the pleiotropic drug resistance transcription factor Pdr3) are able to rearrange their metabolism and accumulate intracellular glucose, glycerol, and inorganic phosphate when grown under glucose or ammonium limitation, in what the authors argue is an alternative defense mechanism and a pre-adaptive response to the loss of the drug resistance genes *PDR3* and *QDR3* (Dikicioglu et al., 2013). These findings were not replicated for either Qdr1 or Qdr2, indicating a wider role of Qdr3 in cellular physiology. However, one of the most significant physiological roles of Qdr3 is probably its involvement in the export and homeostasis of polyamines (Teixeira et al., 2011). Polyamines are essential organic cations that are implicated in the regulation of nucleic acid and protein synthesis, as well as the gating of several ion channels (Cohen, 1998). Unlike in higher eukaryotes, polyamine transport in the yeast *S. cerevisiae* has been characterized in recent years (Igarashi and Kashiwagi, 2010), and four transporters among the DHA1 family were first described as polyamine transporters, Tpo1, Tpo2, Tpo3, and Tpo4 (Tomitori et al., 1999; Albertsen et al., 2003; Uemura et al., 2005). Qdr3 was also found to be involved in the homeostasis of the polyamines spermine and spermidine (but not putrescine), with *QDR3* expression leading to a drop in the intracellular accumulation of spermidine and to a decrease in spermidine-induced disruption of the plasma membrane potential (Teixeira et al., 2011). Interestingly, although both Qdr2 and Qdr3 are determinants of resistance to polyamines and share several of their putative drug substrates (Sá-Correia et al., 2009), Qdr3 has no described role in mono- and divalent cation homeostasis, while Qdr2 is apparently not involved in polyamine transport (Teixeira et al., 2011).

Tpo1-4 confers resistance to toxic concentrations of the polyamines spermine, spermidine and putrescine (Tomitori et al., 2001). Tpo1 is the DHA1 transporter with the widest described range of compounds to which it can confer chemoprotection (Sá-Correia et al., 2009). However, one question that has often been asked is whether Tpo1 confers resistance to all of these unrelated compounds directly or indirectly through its role in polyamine homeostasis. One important contribution to answer this question was the demonstration that Tpo1-mediated spermine and spermidine export plays a role in the response to oxidative stress by changing the intracellular concentrations of these polyamines to control the timing of central components of that response (induction of antioxidant proteins, cell cycle delay, etc.) (Kruger et al., 2013). These observations constitute an important argument in favor of an indirect role of several MDR/MXR-MFS transporters in multidrug and stress resistance, at least partially through an indirect regulation of the stress response machinery via a metabolic control mechanism. Interestingly, although the Hol1 transporter does not have an attributed role in MDR/MXR, specific *HOL1* mutations have been shown to enhance the ability of yeast cells to import histidinol (a precursor of histidine) and mono- and divalent cations (Wright et al., 1996).

#### The DAG family

The DAG family in *S. cerevisiae* includes 16 proteins, encoded by *ATR1*, *AZR1*, *SGE1*, *VBA1*, *VBA2*, *VBA3*, *VBA4*, *VBA5*, *YMR279c*, and *YOR378w* (the DHA2 subfamily, in general less well characterized than the DHA1), *ARN1*, *ARN2*, *SIT*, and *ENB1* (ARN subfamily), *GEX1* and *GEX2* (GEX subfamily) (Table 2) (Sá-Correia et al., 2009; Dias and Sa-Correia, 2013). Unlike the members of the DHA1 family, the majority of the DHA2 proteins have not yet been associated with drug resistance. Only three proteins, Atr1, Azr1, and Sge1, have been shown to provide broad chemoprotection (Sá-Correia et al., 2009). Among them, only Atr1 has a proposed physiological role as the main boron exporter in yeast (Kaya et al., 2009). Conversely, several DHA2 proteins have been assigned substrates of biological significance, but have not been described as resistance determinants. Following the identification of *ATR1* in a genetic screen for boron tolerance (Kaya et al., 2009), *YMR279c*, a paralog of *ATR1*, was also suggested to encode a boron exporter and be involved in boron tolerance, but not *YOR378w*, another paralog of *ATR1* (Bozdag et al., 2011). Interestingly, *ATR1* was also suggested to be involved in the response to DNA replication stress, with its protein abundance being increased following MMS treatment (Tkach et al., 2012). Also, *YOR378w* has been implicated in the TOR pathway in a microarray-based high-throughput screen (Butcher et al., 2006). Overexpression of *YPR378w* was found to significantly increase the sensitivity of yeast cells to rapamycin, the inhibitor of the TOR pathway, with possible implications in multiple nutrient signaling pathways, cell growth and metabolic regulation.

The DHA2 subfamily also includes five transporters that have been postulated as vacuolar basic amino acids permeases, Vba1, Vba2, Vba3, Vba4, and Vba5. Vba1-3 has been shown to mediate the import of histidine and lysine into the vacuole, while Vba2 is also able to catalyze the vacuolar uptake of arginine (Shimazu et al., 2005). Although Vba4 is also localized to the vacuolar membrane and exhibits high sequence similarity to Vba1-3, it has not been shown to be involved in the vacuolar uptake of basic amino acids and its physiological role has not yet been demonstrated. Vba5, although a very close homolog of Vba3, is localized to the plasma membrane instead of the vacuolar membrane, and catalyzes the uptake of lysine and arginine into the cell (Shimazu et al., 2012). Interestingly, although the role of these MDR/MXR-MFS transporters in chemical stress resistance has been investigated, only *VBA5* was associated with MDR, its overexpression leading to increased susceptibility to 4-NQO and quinidine (Shimazu et al., 2005, 2012).

The ARN family, recently found to share an evolutionary root with the DHA2 subfamily and integrated in the newly named DAG family (Dias and Sa-Correia, 2013), encodes four proteins involved in the uptake of siderophore-iron chelates that have been characterized for several years now, Arn1 (Heymann et al., 2000a), Arn2 (Heymann et al., 1999), Sit1 (Lesuisse et al., 1998), and Enb1 (Heymann et al., 2000b). The main natural substrates of Arn2 and Enb1 are the bacterial enterobactin and triacetylfusarinine C, respectively (Heymann et al., 1999, 2000b), with high siderophore substrate specificity, while Arn1 and Sit1 exhibit a broader and overlapping range of siderophore substrates, including several ferrichromes (Lesuisse et al., 1998; Heymann et al., 2000a; Haas et al., 2008). However, other possible roles for these proteins have been proposed. For example, Arn1 was shown to relocalize from the nucleus to the vacuole [although again the localization under control conditions does not match the localization predicted by Huh et al. (2003)] and increase in abundance under DNA replication stress induced by the same agents, suggesting a role in DNA damage response pathways (Tkach et al., 2012).

Although the GEX proteins share a high sequence similarity to the ARN transporters and are dependent on the iron responsive transcription factor Aft2, they are apparently not involved in siderophore transport and have instead a physiological role as glutathione extrusion pumps, mostly localized at the vacuolar membrane but also at the plasma membrane (Dhaoui et al., 2011; Thorsen et al., 2012). Null mutant *gex1gex2* strains are deficient in pH and glutathione homeostasis and are hypersensitive to H_2_O_2_, while overexpression of Gex1 resulted in modulation of the cAMP/PKA and PKC1-MAPK signaling pathways, hinting at a connection between iron, redox equilibrium, and the oxidative stress response (Dhaoui et al., 2011). Furthermore, Gex1 was also suggested to mediate cadmium efflux at the plasma membrane and participate in cadmium detoxification, either alone or as a complex with glutathione (Dhaoui et al., 2011). Given the role of glutathione in heavy metal detoxification and protection against oxidative stress, the natural physiological roles of the GEX proteins might, as previously described for Tpo1 (Kruger et al., 2013), indirectly regulate the stress response machinery, possibly through hierarchical signaling pathways.

### Evolution of the DHA1 and DAG families

Over the past few years, new clues on the evolution of MDR/MXR-MFS genes in yeast species belonging to the sub-phylum *Saccharomycotina* (most commonly known as the Hemiascomycetes) have been obtained, based on the increasing abundance of available genome sequences, which may help to understand several of the issues raised regarding the action of such transporters (Gbelska et al., 2006; Dias et al., 2010; Dias and Sa-Correia, 2013). Phylogenetic and gene neighborhood approaches were combined to reconstruct the evolution of DHA1 genes encoded in the genome of 13 hemiascomycete yeasts (of species *S. cerevisiae, S. bayanus, S. castellii, Candida glabrata, Kluyveromyces polysporus, Zygosaccharomyces rouxii, S. kluyveri, K. waltii, K. thermotolerans, K. lactis, Eremothecium gossypii, Debaryomyces hansenii, Yarrowia lipolytica*), with the identification of 10 main gene lineages (Dias et al., 2010). The construction of a DHA1 phylogenetic tree identified 20 clusters, 11 of which did not contain any *S. cerevisiae* members. The chromosome environments where DHA1 genes reside was found to be remarkably conserved in the hemiascomycete yeast species, with the exception of the lineage containing *FLR1* homologs (Dias et al., 2010). Gene duplication and loss were suggested to be the major forces driving the evolution of the DHA1 protein family (Dias et al., 2010).

The evolution of DAG genes encoded in the genome of 31 hemiascomycetous yeast strains from 25 species (*S. cerevisiae*, *S. paradoxus*, *S. mikatae*, *S. bayanus*, *S. kudriavzevii*, *S. castelli*, *C. glabrata*, *K. polysporus*, *Z. rouxii*, *S. kluyveri*, *K. waltii*, *K. thermotolerans*, *K. lactis*, *E. gossypii*, *Candida albicans*, *Candida dubliniensis*, *Candida tropicalis*, *Candida parapsilosis*, *Lodderomyces elongisporus*, *Candida guilliermondii*, *D. hansenii*, *Pichia stipitis*, *Candida lusitaniae*. *P. pastoris*, *Y. lipolytica*) was also reconstructed (Dias and Sa-Correia, 2013). The phylogenetic tree obtained also identified 20 clusters, with 11 of them lacking *S. cerevisiae* members. Gene neighborhood analysis suggested that gene duplication and loss as well as lateral transfers were the most important driving forces in the evolution of the hemiascomycetous DAG genes (Dias and Sa-Correia, 2013). Lateral gene transfer is proposed to have affected the lineages containing the following *S. cerevisiae* DAG genes: *SGE1, VBA3, VBA5, VBA4, VBA2, GEX1/GEX2, ARN2*, and *YOR378W*. The hypothesis that an abundant number of species-to-species transference of DAG genes has occurred during the evolution of the hemiascomycete yeasts is consistent with the fact that the majority of *S. cerevisiae* DAG genes does not show synteny with the corresponding homologs encoded in early divergent *Saccharomycetaceae* species (Dias and Sa-Correia, 2013). Also, with the exception of *ATR1, YMR279C, AZR1, VBA1*, and *VBA4*, *S. cerevisiae* DAG genes reside in sub-telomeric regions, the preferential chromosomal residence of horizontally acquired genes (Fairhead and Dujon, 2006).

Although the sequences comprised in each phylogenetic cluster for both DHA1 and DAG families are significantly conserved, suggesting that each cluster might contain genes with a specific biological function in common, in several cases we could not find a direct connection between sequence similarity and function. Qdr1 and Qdr2 are good examples, with extremely high amino acid conservation, and yet their main proposed physiological functions (e.g., involvement in spore wall assembly for Qdr1 Lin et al., 2013 and the role in boron extrusion and homeostasis of several cations for Qdr2 Vargas et al., 2007; Rios et al., 2013) are not overlapping. Another example is the ARN transporters and their siderophore substrate specificity (Lesuisse et al., 1998; Heymann et al., 1999, 2000a,b), which cannot be immediately explained by the observed phylogenetic relationships (Figure 1).

Nevertheless, from the consistent retention of DHA1 and DAG genes during the evolution of the Hemiascomycetes, it is likely that the encoded proteins may be important for yeast fitness and survival in the diverse range of ecological niches occupied by these microorganisms in nature. Also, the fact that the ARN and GEX transporters have not been specifically implicated in MDR/MXR (with the exception of a few unexploited phenotypes of altered fitness and gene expression in response to chemical stress, identified in genome-wide screens) further hints at a more complex involvement of these MDR/MXR-MFS transporters in chemoprotection.

## Clues on the functional analysis of DHA1/DAG transporters based on their transcriptional regulation under environmental challenges or stress

During the past few years, much information has been gathered on the transcriptional regulation of the genes encoding DHA1/DAG transporters in *S. cerevisiae*, mostly coming from genome-wide experiments. This information, gathered in the YEASTRACT database (www.yeastract.com) (Teixeira et al., 2006, 2014) highlights interesting hypotheses for the function of the DHA transporters, not only in the multidrug resistance context, but also within the perspective of their physiological roles. It is interesting to note that the regulation of DHA1/DAG genes is far more intricate that initially imagined. Beyond the expected role of MDR transcription factors, a number of stress response and nutrient availability transcription factors have been found, throughout the years, to regulate DHA1/DAG genes. Indeed, the number of regulatory associations with DHA1/DAG genes involving transcription factors virtually not related to MDR is much higher than that involving recognized MDR transcription factors, even when considering only direct regulatory associations, based on DNA-binding evidence. As detailed below, the transcriptional regulation by non-MDR transcription factors has, in a few cases, been seen to be consistent with the physiological role of the DHA1/DAG transporters, suggesting that there is much more to learn from the clues provided by transcription regulation data.

**Figure 2:**
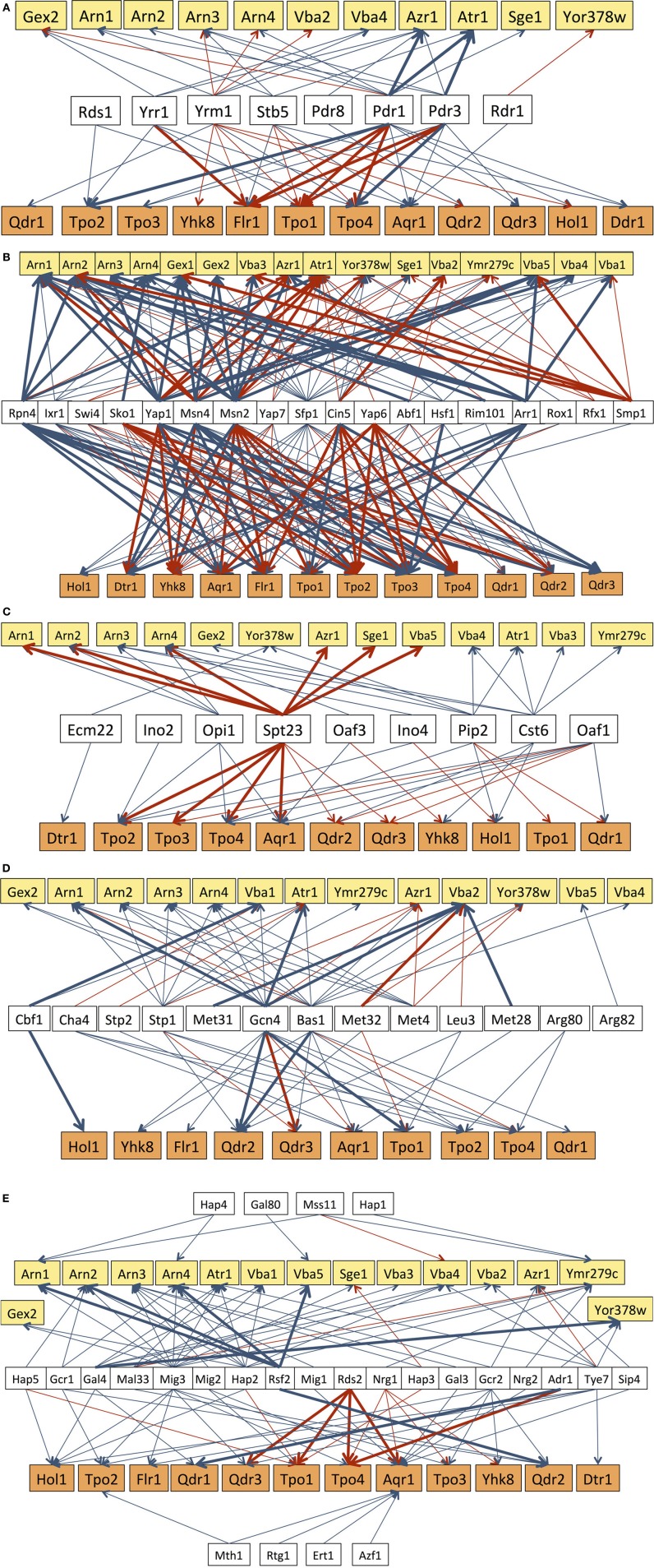
**Transcriptional regulatory networks that control the expression of DHA/DAG genes, considering the subgroups of transcription factors known to be involved in multidrug resistance (A), in stress response (B), lipid metabolism (C), or in the response to amino acid (D) and carbon source (E) availability**. The displayed regulatory associations are according to the data present in the Yeastract database (www.yeastract.com, Teixeira et al., 2006, 2014), as of February 2014. Arrows indicate the experimental basis of the documented regulatory associations, either expression evidence (blue arrows) or DNA-binding evidence (red arrows). Bold arrows indicate the cases in which the regulatory association was found to take place in response to the environmental condition considered in each subgroup of transcription factors.

In what concerns multidrug resistance, 23 out of the 28 DHA1/DAG transporters in *S. cerevisiae* are known to be regulated by at least one MDR-related transcription factor, specifically, all of the DHA1 transporters and nearly 70% of the DAG transporters (Figure 2A). Remarkably, Pdr1 was found to regulate nearly 60% of the DHA1/DAG genes. Transporters that have been more clearly linked to resistance to a wider range of drugs are indeed those that are regulated by a higher number of MDR transcription factors. This is the case for *FLR1* and *TPO1*, which are regulated by Yrr1, Yrm1, Stb5, Pdr1 and Pdr3. In particular, the *FLR1* regulation is a paradigmatic case of study. Its regulation is highly complex, being controlled, for example, under mancozeb stress by at least four transcription factors (Teixeira et al., 2008), two of which, Pdr3 and Yrr1, are linked to multidrug resistance, and two are involved in stress response, Yap1 and Rpn4. A more detailed analysis of this network, based on a systems biology approach, has highlighted even further the possible influence of a fifth transcription factor (Teixeira et al., 2010), which remains unidentified. It is also interesting to point out the cases of Hol1, Vba2, Vba4, Yor378w, Arn1-4 and Gex1 which, despite the absence of evidence for a role in MDR/MXR, are indeed regulated by MDR transcription factors (Figure 2A).

All of the DHA1/DAG genes have been found to be regulated by, at least, seven stress related transcription factors, with the transcription factors Sfp1 (86%), Msn2 (71%), Msn4 (64%), and Yap1 (61%) regulating more than 60% of the DHA1/DAG genes (Figure 2B). Msn2 and Msn4 are the key regulators of the general stress response, while Yap1 is involved in the oxidative stress response and also in specific cases of drug resistance, and Sfp1 is involved in ribosome biogenesis and, indirectly, in resistance to many stress conditions. The regulatory associations involving Rpn4, a transcription factor that controls proteosomal genes and that is implicated in the oxidative stress response and DNA repair (Jelinsky et al., 2000; Karpov et al., 2013), in particular induced by the genotoxic agent MMS (Spasskaya et al., 2014), are also of interest. Rpn4 has been shown to regulate 43% of all DHA1/DAG genes, including the 4 genes (*ARN1*, *ATR1*, *AQR1*, and *FLR1*) whose expression and/or localization was found to change in response to MMS treatment leading to replicative stress (Tkach et al., 2012). Many more stress related transcription factors, although falling out of this analysis since they only regulate less than six of the DHA1/DAG genes, may also provide interesting clues on the function of this family of transporters. This is the case, for example, for Haa1, a key regulator of acetic acid stress response (Fernandes et al., 2005), which has been reported to control the expression of *TPO2* and *TPO3* in response to acetic acid (Mira et al., 2010, 2011), acetaldehyde (Aranda and del Olmo, 2004) and copper stress (Keller et al., 2001), and also to affect the basal expression of *YMR279c* (Reimand et al., 2010).

All but one of the DHA1/DAG genes are also regulated by nutrient availability transcription factors. Thirteen transcription factors involved in the response to amino acid availability have been found to regulate 70% of the DHA1/DAG genes. Among these transcription factors, Gcn4 and Bas1 stand out as the two that regulate the majority (more than 60%) of these genes (Figure 2C). Gcn4 is an activator of amino acid biosynthetic genes, in response to amino acid starvation, while Bas1 regulates the expression of genes in the purine and histidine biosynthesis pathways. In some cases, a clear link between the function of the DHA1/DAG genes and amino acid homeostasis has been established. This is the case for Aqr1, proposed to play a role in the extrusion of amino acids, particularly homoserine and threonine (Velasco et al., 2004). The Vba transporters have also been described as vacuolar membrane amino acid transporters (Shimazu et al., 2005), while Qdr2 has been shown to affect amino acid homeostasis, especially in cells experiencing leucine limitation (Vargas et al., 2007). The remaining DHA1/DAG transporters have no described link to amino acid homeostasis. However, the fact that they are regulated, at the transcriptional level, by several transcription factors controlling amino acid homeostasis, suggests that they may also play a role in this context. This appears to be especially the case for Tpo2, Tpo4, and Atr1, which are each regulated by five of these transcription factors.

Twenty-six transcription factors linked to carbon source availability have been shown to regulate at least one DHA1/DAG gene. It is interesting to point out that the transcription factors of this group that regulate a higher number of DHA1/DAG genes are Mig3 (61%), Hap2 (36%), and Tye7 (32%). Mig3 plays a major role in carbon catabolite repression and ethanol response, but is also involved in the response to toxic agents, while Hap2 is one of the key regulators of respiration and Tye7 is an activator of glycolytic gene expression. Among the 27 DHA genes regulated by carbon source related transcription factors, eight are regulated by five or more of these transcription factors, including Atr1 (5 TFs), Vba4 (5 TFs), Arn3 (6 TFs), Arn4 (5 TFs), Ymr279c (7 TFs), Hol1 (5 TFs), Tpo1 (5 TFs), and Aqr1 (12 TFs) (Figure 2C). Very little is known however on the role of DHA1/DAG transporters in carbon metabolism, although recent studies have suggested a respiratory deficiency of *qdr3* null mutants under glucose deprivation (Dikicioglu et al., 2008).

Another analysis of interest concerns the regulation involving lipid metabolism-related transcription factors. The overall role of ABC transporters in membrane lipid homeostasis, and consequently in stress tolerance and drug resistance, has been widely discussed in recent years (Panwar et al., 2008; Shahi and Moye-Rowley, 2009; Peetla et al., 2013). In fact, fluctuations in membrane composition are known to alter drug distribution and accumulation, and also to affect the localization and function of MDR pumps. The involvement of MDR/MXR transporters in the regulation of membrane composition has been shown to provide an important contribution to MDR/MXR resistance (Shahi and Moye-Rowley, 2009). Although the same type of association has never been established for MDR/MXR-MFS transporters, the fact that 9 lipid metabolism-related transcription factors regulate 24 out of the 28 might be indicative of a similar, still unidentified, role for MDR/MXR-MFS transporters in membrane lipid homeostasis or lipid-based signaling. Particularly noteworthy are the transcription factors Spt23, Cst6, and Pip2 which regulate 46, 32, and 32% of the total number of DHA/DAG genes. Spt23 is involved in the regulation of the single yeast fatty acid desaturase Ole1, while Cst6 and Pip2 are regulators of oleate responsive genes. Interestingly, the action of Spt23 in its target DHA/DAG genes was found to occur under heat shock (Auld et al., 2006), while that of Pip2 upon *ARN2*, *TPO1*, and *TPO4* was registered in cells growing on oleate as carbon source (Smith et al., 2007).

It also appears noteworthy that many of the regulatory associations found so far to involve DHA1/DAG genes have been reported under non-stress conditions. Figures 2A–E highlight in bold the cases in which the reported association is known to take place under the stress in which the corresponding group of transcription factors is known to act. This observation suggests that the control of the basal expression of the DHA1/DAG genes is also crucial for the maintenance of cellular homeostasis, even under non-stressing conditions, strongly pointing out that these so-called “drug transporters” must serve much more important and wider roles in cell physiology, which are still far from being fully understood.

Finally, it is important to point out that these analyses are based on Yeastract information “as is.” This is relevant because the interactions reported therein come from different sources and rely on different experimental approaches. Interactions can be based on indirect (e.g., transcriptomic analyses) or direct (e.g., ChIP data) evidences, and the number of independent studies supporting each regulatory interaction can vary greatly. Here we have opted to show all interactions described for each TF-target gene considered, although we distinguish between expression evidence and DNA-binding evidence (red arrows vs. blue arrows, respectively; Figures 2A–E). Therefore, the regulatory networks that are discussed here should be considered with this in mind, and the level of confidence of each interaction can be assessed in Yeastract by the readers whenever relevant.

## Future perspectives and concluding remarks

Yeast MDR/MXR-MFS transporters have been traditionally characterized as transporters of a significantly wide spectrum of structurally unrelated cytotoxic compounds. However, their prevalence and apparent redundancy has led to a longstanding debate regarding the natural function of these transporters in the cell, and on whether their action in the context of chemoprotection and stress resistance is direct, indirect or a combination of both (Sá-Correia et al., 2009). Mounting evidence seems to favor the latter hypothesis, and even for ABC transporters, the paradigmatic drug pumps that include the well-characterized human P-glycoprotein (Kartner et al., 1983), there is an increasing acknowledgement of the indirect contribution of these transporters to the resistant phenotype through the control of plasma membrane potential and membrane lipid homeostasis, which in turn can affect drug delivery to intracellular targets and its accumulation (see Prasad and Panwar, 2004; Panwar et al., 2008; Shahi and Moye-Rowley, 2009; Peetla et al., 2013) for reviews on this topic). The experimental evidence reviewed here regarding the yeast MDR/MXR-MFS transporters highlights a significant number of recent studies unveiling new and unexpectedly complex roles of these transporters that are shown to contribute to their role in MDR/MXR, in particular through metabolic regulation of the stress response machinery, in addition to the previously proposed action on the control of membrane potential and/or internal pH (Roepe et al., 1996; Sá-Correia et al., 2009). Interestingly, phylogenetic analyses of the DHA1 and DHA2 subfamilies in other hemiascomycete yeasts have identified several functionally characterized transporters presumably involved in the MDR/MXR phenomenon (Gbelska et al., 2006; Dias et al., 2010; Dias and Sa-Correia, 2013). These include CgTpo3, which plays a role in polyamine homeostasis and azole drug resistance (Costa et al., 2014), mirroring its *S. cerevisiae* homolog Tpo3; Nag3, and Nag4, which catalyze the uptake of N-acetylglucosamine in *Candida albicans* and are also involved in drug sensitivity and virulence (Yamada-Okabe and Yamada-Okabe, 2002; Sengupta and Datta, 2003; Wendland et al., 2009); Ffz1 and Ffz2, two characterized importers of fructose in *Zygosaccharomyces rouxii* (Leandro et al., 2011) (and *Z. bailli* for Ffz1, Pina et al., 2004) with no known role in MDR/MXR; and Knq1, a *Kluyveromyces lactis* transporter that is involved in iron homeostasis, drug resistance and oxidative stress response under the control of KlYap1 (Takacova et al., 2004; Imrichova et al., 2005; Marchi et al., 2007), the *K. lactis* homolog of *S. cerevisiae*'s Yap1. Interestingly, although *KNQ1* shares some sequence identity with *ATR1*, there is no close homolog in *S. cerevisiae*, with the exception of *S. cerevisiae* JAY-291, a fully sequenced strain that is widely used in bioethanol production (Argueso et al., 2009). The functional characterization of DHA proteins identified in other yeast species that no longer have a close homolog encoded in the genome of *S. cerevisiae* might also contribute to elucidate the physiological relevance of these transporters.

The extension of the current knowledge on yeast MDR/MXR transporters to pathogenic yeasts, in particular *Candida* species, is another crucial area that in the future is expected to contribute to the management of the rising number of drug resistant fungal infections. However, the MFS family of multidrug transporters in *Candida* species remains largely uncharacterized, and there is in general a much stronger association between clinical acquisition of antifungal drug resistance and the expression of ABC drug pumps (Gaur et al., 2008; Cannon et al., 2009). The first MDR/MXR-MFS transporter to be characterized from a pathogenic fungus was the paradigmatic Mdr1 protein in *C. albicans*, a close homolog of *S. cerevisiae* Flr1 and a determinant of resistance to azole drugs, benomyl, methotrexate *etc*. (Fling et al., 1991; Ben-Yaacov et al., 1994). More recently, *C. glabrata* homologs of DHA1 Qdr2 (Costa et al., 2013a), Aqr1 (Costa et al., 2013b), and Tpo3 (Costa et al., 2014) have been implicated in antifungal drug resistance, while the homolog of Aqr1 and Tpo3 were further shown to have a role in acetic acid resistance and polyamine homeostasis, respectively (Costa et al., 2013b, 2014). In phytopathogenic fungi, which constitute an important problem in crop protection, MDR/MXR-MFS transporters such as BcMfs1 from *Botrytis cinerea* (Hayashi et al., 2002) and MgMfs1 from *Mycosphaerella graminicola* (Roohparvar et al., 2007, 2008) have also been shown to be involved in resistance to natural plant toxins and fungicides. Altogether, the role of many DHA transporters from fungal species was found to, at least partially, mirror their *S. cerevisiae* homologs, highlighting the relevance of studies in the model yeast to elucidate the regulation and functional role of these transporters in other species.

Further characterization of drug resistance MFS proteins in pathogenic yeasts or phytopathogenic fungi will benefit from studies in *S. cerevisiae*, in particular from transcriptional data that might help elucidate the putative physiological roles of these transporters. Although it remains to be clarified if the complex transcriptional networks previously described for *S. cerevisiae* have parallels in other yeasts, recent findings regarding the transcriptional regulation of *ScFLR1* orthologs in *C. albicans* and *C. glabrata* (*CaMDR1* and *CgFLR1*, respectively) show that it is at least partially similar to that in *S. cerevisiae* (Gulshan and Moye-Rowley, 2007), whereas the transcriptional control of *CgQdr2* and *CgTpo3* (Costa et al., 2013a, 2014) appears to differ from that of their *S. cerevisiae* counterparts. The recent finding on MDR/MXR-MFS transporters in *Candida* species will be the focus of another review in this research topic (see Costa et al. in this Research Topic).

The knowledge gathered in yeast MDR/MXR-MFS transporters can also be extrapolated to other systems and less accessible higher eukaryotes such as plants. Abiotic stress is an important contributor to loss of plant productivity worldwide under agricultural conditions. The clarification of the mechanisms underlying MDR/MXR in plants will be instrumental in paving the way for the development of efficient strategies to improve crop tolerance to abiotic stress, in particular regarding the potential role of MDR/MXR transporters in the plant response to heavy-metal contamination, high salinity, pesticides, and other xenobiotic compounds. Considerable progress has been made on the characterization of plant ABC transporters, which have been shown to play a pivotal role in numerous physiological processes (van den Brule and Smart, 2002), however the functional significance of MFS transporter genes in plants remains largely unknown (discussed elsewhere in this research topic). Given the conservation of transport mechanisms from *S. cerevisiae* to higher eukaryotes, Tpo1 homologs encoding putative plasma membrane MFS transporters from the plant model *Arabidopsis thaliana* were analyzed to uncover new roles for these transporters in plant abiotic stress tolerance (Cabrito et al., 2009). This led to the identification of Zifl1, the first eukaryotic MFS transporter identified as a multidrug resistance determinant. *ZIFL1* transcription levels were found to be elevated in plants treated with the herbicide 2,4-D, and heterologous expression in yeast complemented the sensitivity phenotype in *tpo1* null mutant cells stressed with 2,4-D (Cabrito et al., 2009). This study is a proof of concept that validated the combined use of the two model systems, *S. cerevisiae* and *A. thaliana*, to uncover novel roles for plant MFS transporters, which may open new avenues for the development of efficient strategies to improve crop productivity or decontaminate polluted soils.

In recent years, the use of genome-wide approaches in the model yeast *S. cerevisiae* has significantly increased our knowledge on the evolution, physiology and regulation of MDR/MXR-MFS transporters, contributing to elucidate the mystery of the redundancy and promiscuity of these transporters and their physiological relevance. In the near future, more specialized global approaches such as metabolomics and lipidomics are expected to expand on the recent findings here reviewed, in particular regarding the possible indirect role of these transporters in the regulation of the stress response, either through metabolic regulation, alteration of the plasma membrane composition, or other mechanisms. The exploitation of high-throughput technologies and the knowledge gathered in yeast can prove invaluable to guide studies in pathogenic fungi, to develop more robust yeast strains for biotechnological applications, and to improve crop protection, with promising repercussions from medicine to biotechnology and agriculture.

### Conflict of interest statement

The authors declare that the research was conducted in the absence of any commercial or financial relationships that could be construed as a potential conflict of interest.
